# What do consumer and providers view as important for integrated care? A qualitative study

**DOI:** 10.1186/s12913-022-08997-x

**Published:** 2023-01-04

**Authors:** Ann Carrigan, Natalie Roberts, Robyn Clay-Williams, Peter Hibbert, Elizabeth Austin, Diana Fajardo Pulido, Isabelle Meulenbroeks, Hoa Mi Nguyen, Mitchell Sarkies, Sarah Hatem, Katherine Maka, Graeme Loy, Jeffrey Braithwaite

**Affiliations:** 1 Australian Institute of Health Innovation, Centre for Healthcare Resilience and Implementation Science, Macquarie University, Sydney, New South Wales Australia; 2 Centre for Elite Performance, Expertise & Training, Macquarie University, Sydney, NSW Australia; 3 IIMPACT in Health, Allied Health and Human Performance, University of South Australia, Adelaide, SA Australia; 4 Western Sydney Local Health District, Sydney, New South Wales Australia

**Keywords:** Multidisciplinary team, Healthcare, Interdisciplinary, Consumer satisfaction, Provider satisfaction

## Abstract

**Background:**

Integrated care is a model recognised internationally, however, there is limited evidence about its usability in the community. This study aimed to elicit community and provider views about integrated care and how implementation could meet their healthcare needs in a new hospital.

**Methods:**

Using a qualitative approach, consumer and provider views on the strengths, barriers and enablers for integrated care were collected via a series of online workshops and supplementary interviews.

**Results:**

A total of 22 consumers and 49 providers participated in 11 focus groups; all perceived integrated care to be an accessible and efficient model that offers a high level of care which enhanced staff and patient well-being. Providers expressed concerns about longer waiting times and safety risks associated with communication gaps and insufficient staff. Enablers include supporting consumers in navigating the integrated care process, co-ordinating and integrating primary care into the model as well as centralising patient electronic medical records.

**Discussion:**

Primary, tertiary and community linkages are key for integrated care. Successful interoperability of services and networks requires an investment in resources and infrastructure to build the capability for providers to seamlessly access information at all points along the patient pathway.

**Conclusion:**

Integrated care is perceived by consumers and providers to be a flexible and patient-focused model of healthcare that offers benefits for a hospital of the future.

**Supplementary Information:**

The online version contains supplementary material available at 10.1186/s12913-022-08997-x.

## Background

**Fig. 1:**
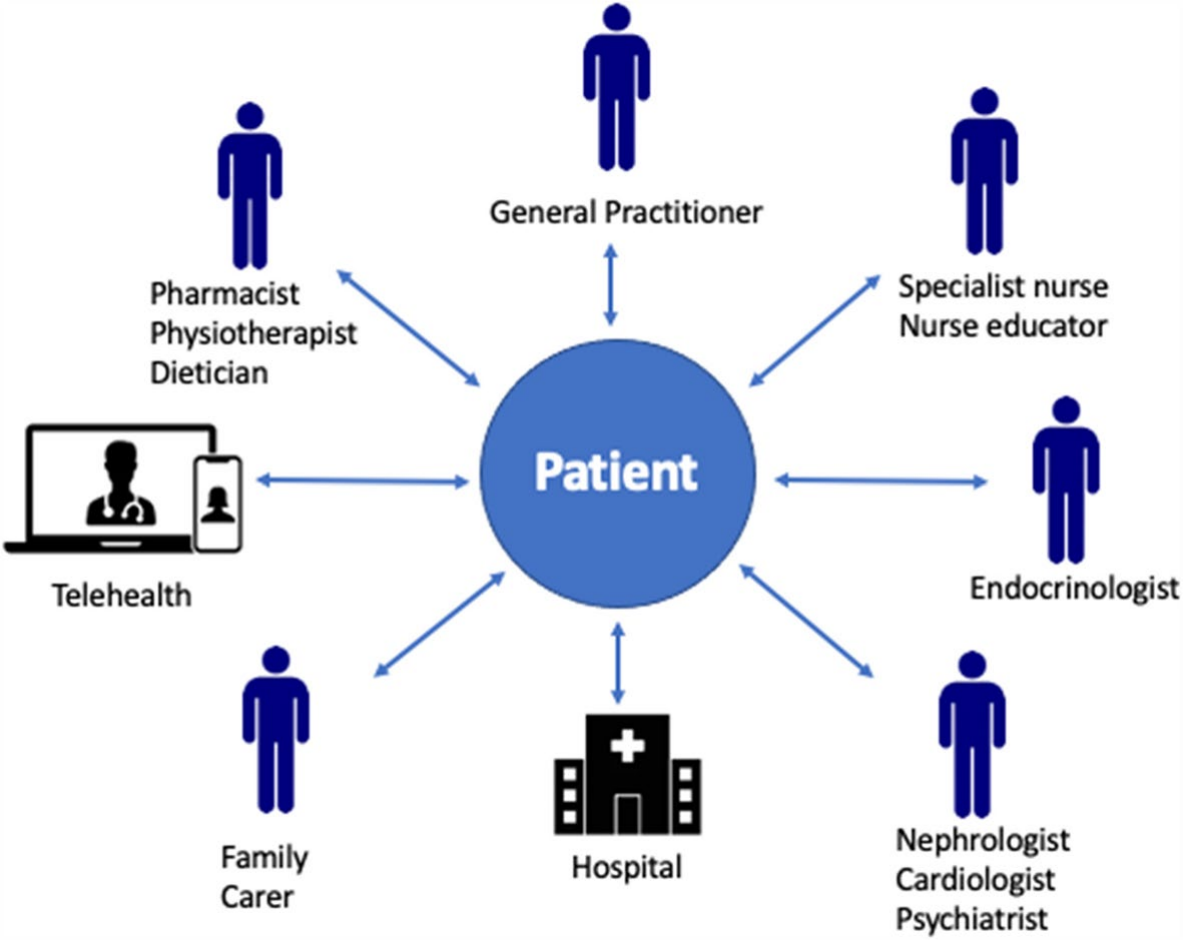
Example of an integrated care model in the context of a chronic disease (adapted from Rocca et al.) [6]

The delivery of quality healthcare is rapidly transforming from a traditional model of a single facility where care is siloed according to organ disease or injury, to alternative models that support patient choice, greater flexibility, broad and community focused [1]. These changes are driven by in part by increasing demand brought about by the COVID-19 global pandemic and also the higher prevalence of multi-morbidities and chronic diseases experienced globally [1], a need for patient-centred, holistic healthcare models [2], and increasing availability of cost-effective care alternatives often delivered in the community [3]. In Australia, healthcare has been identified as fragmented, largely due to a lack of national co-ordination of healthcare services across states [4]. Integrated care, a globally accepted model, involves patient centred care via a single point-of-entry and typically involves merging or increasing collaboration among care services, organisations and providers [5] (See Fig. 1). Integrated care may offer a solution to these systemic problems.

The reported benefits of integrated care include a reduced number of patient appointments [7], improved continuity of care [7], better co-ordination of services [8, 9], more personalised care [9], reduced cost [10], improved quality of life [11], and improved patient outcomes [12]. Some challenges have been identified with implementing this model, including a lack of investment in infrastructure and organisational management [13], and compromised care as a result of communication failures [14–16]. For example, one study found 61% of sentinel events were due to communication failures among teams [17]. Despite these challenges, healthcare facilities are increasingly integrating care [13] that incorporates consumer-focused care principles [18, 19].

Integrated care has been implemented across a wide range of health conditions that include chronic kidney disease (CKD) [20], diabetes, heart failure, depression, and chronic obstructive pulmonary disease (COPD) [21]. Integrated care has a significant effect on decreasing heart failure readmission rates [22, 23], and reducing ED visits for COPD patients [21]. Pharmacist involvement in integrated care has been shown to reduce hospitalisation visits for patients with heart failure and improve health related quality of life, but offers no improvements in self care [23]. Positive effects have been found for hip fracture including improvement of physical and health outcomes, and increased mobility [24]. Improvements in blood pressure of CKD patients and heart rate and oxygen saturation in patients with COPD [21] have also been reported.

While integrated care is being adopted globally, evidence obtained from consumers and providers about their experiences and perceptions of the model, and how it might be applied in the design of a new healthcare facility, is lacking. The aims of this study were to elicit consumers’ and providers’ views about models of care for the design of a proposed new healthcare facility in a large, diverse catchment in New South Wales, Australia, but with generalised application of the findings to similar health systems internationally. In preparation for the study, we completed grey and academic literature reviews, which identified six innovative models of care (ambulatory care, digital hospital, hospital in the home, integrated care, virtual care, and specialist hospital) [25]. The current paper reports the consumer and provider views about one of the six innovative models of care: integrated care.

## Methods

The study methods are described in detail in Carrigan et al. [26] Key aspects of the methods relevant to integrated care are outlined below.

### Study design and setting

We undertook a qualitative study of consumer and provider needs, views and preferences in relation to the integrated model of care delivery for a new healthcare facility. We collected quantitative data via a short demographic questionnaire to inform the design of a series of facilitator-coordinated workshops where qualitative data was collected (e.g., provide role where nurses were allocated to one focus group). One participant opted to participate in follow-up interview to provide additional feedback on integrated care. Workshops and supplementary interview were conducted online due to COVID-19 restrictions in place at the time.

### Procedures

#### Recruitment

Consumer participants included residents and patient representatives aged 18 years and over, who were living within the new health facility catchment area. The catchment was defined by the local health district’s (LHD) planning team on 16th July 2021, and included 49 suburbs in Sydney, New South Wales, Australia. A flyer was disseminated through email lists within the LHD’s network, as well as local newspapers and via the LHD Facebook page. Providers aged 18 years and over who worked in the catchment of the new hospital were recruited via LHD email lists and included healthcare professionals, management, and support staff.

The study invitation included a link to an online expression of interest (EOI) questionnaire that captured demographic data hosted on REDCap [27]. Demographic data for all participants included age, gender, residential suburb, and ethnicity. The providers were also asked to indicate their role and specialty, and the consumers were asked for pertinent health information such as whether they have a chronic health condition. After each workshop, those who expressed interest were contacted for a follow-up interview to expand on their focus group responses.

### Data collection

A description of the integrated model of care with an associated patient scenario, and questions tailored for integrated care, were developed based on a common disease identified in the community, Type II diabetes. Focus group guides were created to take consumers through a series of discussions to consider the model’s strengths and weaknesses, enablers, usability and safety for themselves and people in their care (Additional file 1 Appendix 1). Provider group facilitation guides were created to elicit provider views about barriers and enablers associated with the model from their own and their patients’ perspectives (Additional file 2 Appendix 2). Each focus group was facilitated by two researchers; one researcher posed questions to the group, while a second researcher recorded notes. Each research pair met following the focus group and together reviewed the notes, to ensure that the recorded data accurately reflected what was said in the focus group. Rotation of researchers over the course of data collection ensured variation in pairing across the focus groups, and this helped to reduce the risk of bias. The integrated care model was presented at 11 two-hour workshops, five for consumers and six for providers. One of the provider workshops was specifically conducted for primary health care providers (General Practitioners; GPs). One consumer volunteered for a follow-up interview (Additional file 3 Appendix 3).

After introducing the workshop, facilitators described the integrated care model and presented the scenario:

*Steve is a 50-year-old male with Type II diabetes who is obese and smokes a packet of cigarettes a day. He is having trouble walking so visits his local Emergency Department were he sees a General Practitioner (GP), who has a practice in an office next to the Emergency Department. The GP diagnoses a foot ulcer and identifies that Steve requires a full review of his care. Steve will be looked after in hospital by a multidisciplinary team of healthcare professionals (*e.g.*, endocrinologist, ulcer team, nutritionist) using an electronic medical record system for communication.*

Researchers (one scribe and one facilitator), and participants were then allocated to smaller online focus groups (up to five people). Within each group, the researchers made notes, facilitated discussion, and asked probing questions. Audio-recording devices, and researcher notes were used to capture the content of discussions. For the interview, participants were asked to expand upon topics of interest that were identified in the focus groups.

### Data analysis

Data was collected sequentially and analysed separately. The demographic and health related data from the demographic questionnaire, were descriptively analysed using SPSS V.22.0 [28]. Qualitative consumer and provider workshop and interview data for the model were merged into two aggregated, narrative summaries, one each for consumers and providers. All participants were de-identified, and any identifiable features of the experiences or personal details shared in the group were changed to protect anonymity (e.g., if a unique service or practitioner was mentioned or features of the disease which identifies the patient). Following each focus group, handwritten notes were reviewed and recordings used to refine the notes if clarification needed. Aggregated data sets from hand-written notes were then analysed separately for consumers and providers. Data were thematically analysed independently using an open coding process by two members of the research team (AC, NR) [29]. The researchers met regularly to share results of their initial coding and together synthesised these codes into a series of broad themes. Examples include resources, and patient wellbeing. Several sub-themes were identified and agreed that characterised the expectations and needs of the consumer members and health providers who were part of the new health facility catchment. Regular meetings took place between the research team to ensure intercoder reliability.

## Results

### Description of participants

Twenty-two consumers from across the geographical catchment participated in six workshops. One volunteered for a follow up interview. Forty-nine providers from a diverse range of professional roles and workplaces in the health catchment participated in five workshops. Their age and gender distribution are summarised in Table 1.

**Table 1:** Consumer and provider participant demographics

	Consumer (***n***)	Provider (***n***)
Gender		
Male	9	15
Female	13	34
Age		
Under 30	3	10
31 to 45	5	19
46 to 60	11	15
Over 61	2	5
Prefer not to say	1	0

### Consumers

Consumers reported experiencing health conditions that were a spread across the major physiology systems, with most consumers having experienced cardiac or bone related conditions. This was representative of the catchment where chest pain, heart failure and acute myocardial infarction are listed among the five most common causes of hospitalisation [11].

All the consumers were proficient in English with 23% speaking another language at home such as Punjabi (5%) and Hindi (5%). Although most of the consumers identified as Australian (69%), there was evidence of ethnic diversity such as Indian (11%), European (8%), Asian (4%).

### Providers

**Fig. 2:**
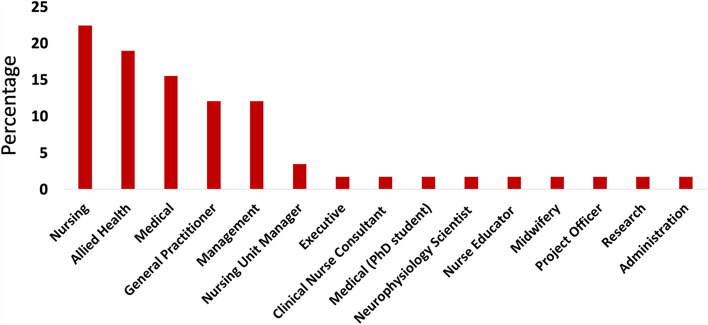
Distribution of provider roles of participants

Forty-seven percent of the providers worked in the LHD while the remaining 53% worked in areas outside of the LHD but resided in the new hospital catchment. The providers worked in a variety of professional roles including nursing, allied health, medical, general practice and administration. Allied health professionals included physiotherapists and speech pathologists (Fig. 2).

The providers reported having a diverse range of specialist qualifications with most practicing in a speciality such as psychiatry (25%), bone (16%), lung (15%), abdominal (13%), heart (12%), postnatal depression (10%), or renal dialysis (9%).

All the providers were proficient in English with 37% speaking another language at home such as Mandarin (17%) and Hindi (11%). Although most of the providers identified as Australian (79%), there was evidence of considerable ethnic diversity such as Asian (18%) and Indian (6%).

### Qualitative results

Results from the qualitative workshops and interview are reported under the overarching topics of *enablers and opportunities;* and *barriers, challenges and risks.* Where common themes exist, reporting of the consumer and provider data are combined under the one heading. Unique themes that were identified are reported for each.

### Enablers and opportunities

For consumers, five common themes emerged to support integrated care: *systems and processes, co-ordination and communication, people*, and *resources and infrastructure,* and *accessibility.* Providers included a sixth theme – *education*.

#### Systems and processes

Around half of the consumers and half of the providers identified the importance of integrating information and communication technologies (ICT) including electronic medical records (EMRs) to facilitate communication between GPs, hospital clinicians and administration, and patients. This may require providing GPs with remote access to hospital ICT systems. A few consumers felt that having an on call, 24-hour team to ensure continual care was important.

Additionally, some providers noted the importance of linking imaging and laboratory testing into the ICTs. The ICTs enable seamless patient reviews, follow up care and co-ordination. Many providers noted that more succinct discharge summaries that adhere to national guidelines and standards are desirable. Several consumers mentioned the need for the model to be evaluated to ensure the model is achieving the desired outcomes.

*The model needs to be evaluated to make sure the model is operating the way it should [and to ensure there are] checks and balances throughout the care plan process* [Consumer 5 workshop 2)].

#### Co-ordination and communication

Co-ordination and communication emerged as a strong theme for consumers and providers. Providers highlighted the importance of team cohesiveness for successful implementation of the model.

*Need to have people who work together regularly [for effective integrated care]* [Provider 3 workshop 2].

*[For] multidisciplinary teams, physical proximity of the team is essential as it really facilitates communication* [Provider 2 workshop 4].

Care co-ordination was portrayed as crucial, with clear roles delineated for each team member. For example, the importance of a central person to facilitate the scheduling of appointments, patient transport, and discharge planning was mentioned to ensure that the patient does not get lost in the system when they return home. Many providers highlighted the role of the GP to lead the care and co-ordinate continuity and adequate after hospital care, and felt that integrated care needed planning prior to patient discharge. Consumers also highlighted the importance of having a patient navigator to ensure they do not “fall through the cracks” in the system.

*[There needs to be a] patient advocate or carer past and present to ensure wrong decisions aren’t made* [Consumer workshop 6].

Communication enablers include having regular multidisciplinary team (MDT) meetings, communication via the EMR, forums to discuss next steps and concerns, and a culture where nurses and allied health professionals will prompt doctors if anything is missed.

*Communication among multidisciplinary teams to look after the patient in a holistic manner [is paramount]* [Provider 8 workshop 4].

*Often the nurses and allied health professionals will prompt doctors if anything is missed. Need to have people who work together regularly* [Provider 3 workshop 2].

*One team takes responsibility and leads the care and communicates with other teams. For example, a GP* [Provider 2 workshop 8].

#### People

Participants identified skilled staff, as well as patient advocates and case navigators or co-ordinators as essential enablers of seamless integrated care, including providing a vital link between step-up and step-down care.

*A patient advocate or carer past and present [is needed] to ensure wrong decisions aren’t made* [Consumer 5 workshop 6].

*A number of different professions with different opinions [are needed] to ensure all gaps and bases are covered* [Consumer 1 workshop 2].

A few providers noted that nurses are the lynchpin for integrated care. Many noted that skilled staff such as pharmacists, social and mental health workers, and others from the community could be included in the model. There was a suggestion that GPs be funded to provide the link for integrated care with outpatient departments and nurses for co-ordinated care.

#### Resources and infrastructure

Secure ICT infrastructure and support, that is accessible to everyone, with centralised and universal patient records were noted by consumers and providers as essential for care integration.

*If a multidisciplinary team extends beyond the hospital, there needs to be a mechanism of communication between these providers* [Provider 1 workshop 4].

Additionally, consumers and providers reported that telehealth infrastructure to support virtual appointments was desirable.

Several noted that adequate supply of resources such as rooms, monitors, internet, staff, and equipment were needed to maintain the model. Design and location of the integrated care facility was highlighted by many as important for relationship building and facilitating ‘corridor’ conversations.

*A properly designed outpatient department is equally important and acts as the middleman between acute care and the community* [Provider 2 workshop 4].

#### Accessibility

Many participants noted that ICT support needs to be integrated with a central directory to ensure that all services are linked. Consumers identified that access to transport, including public services such as buses and private services, and affordable parking were also important, and that this support was providers out of regular work hours.

*[There needs to be a] 24 hour team on call* [Consumer 2 workshop 4].

Providers suggested providing EMR access and hospital admitting rights to GPs as well as evaluating digital literacy of staff and patients. For example, assessing consumer digital literacy to communicate with providers via a telephone or smart device will help to may identify system weaknesses and offer an additional safety net.

#### Education

Patient knowledge of the model coupled with an understanding of how to navigate the health system were identified as key enablers. Specifically, it was considered vital for consumers to know what to do if something unexpected happens in relation to their health condition when at home. It was also highlighted that care providers need to keep abreast of changes in technology. Providers felt that community education had a part to play in building relationships that lead to better care.

*Establishing relationships with people outside the hospital is really important to patient care. Previously this was achieved through education days and seminars with the community. This is an important medium for casual communication and closing feedback loops on the patients and long-term care outcomes. Sometimes education also goes both ways* [Provider 1 workshop 4].

### Barriers, challenges and risks

For consumers and providers, the same five main themes emerged as barriers, challenges, and risks associated with integrated care: *co-ordination and communication, resources and infrastructure, accessibility, skills and abilities, patient factors and well-being* and *safety and risks.*

#### Co-ordination and communication

Consumers identified that lack of care team co-ordination and communication may lead to a patient “falling through the cracks”, especially if they have no centralised support person or navigator. Many providers identified risks associated with transitioning between care organisations, particularly out of hours. Several providers felt that there were communication risks relating to patient data transmission between providers, especially if technology was not available.

Providers reported that it is difficult to make staff accountable for their actions when there is a large team involved, especially if there is a high staff turnover; there is no clear and concise ownership of the patient, or no role delineation. They felt that teams need a clearly defined way to determine who is leading or the primary carer, otherwise responsibility may be shifted. The need for structured staffing processes such as team meetings to support the model was strongly felt by several providers.

*There is no substitute for face to face and multidisciplinary team meetings even if there is an electronic medical system* [Provider 1 workshop 4].

Some described a reluctance of staff to take responsibility due to the phenomena of “social loafing”, whereby individuals in large teams feel that their identity is sufficiently protected that they can safely let others do the work. Some were concerned about people assuming another team is looking after the patient in the instance of an emergency when there is no admitting team, and it was difficult to make staff accountable when there is a big team behind it.

*Need a clearly defined way to determine who is leading or the primary carer, otherwise responsibility may be shifted* [Provider 2 workshop 6].

Also, if staff do not have a clear understanding of how the integrated care model is expected to work, communication barriers combined with poor understanding of how care is delivered across the team could leave the patient with unclear or conflicting advice.

*As long as the definition of integrated care is true to the model [the risk will be acceptable. It is important] that it doesn’t just become siloed clinicians working in the same location. They actually need to work together* [Provider 7 workshop 2].

Some providers were concerned about the continuity of care during weekends, weekdays, community care and inpatient services and that timing and co-ordination was important.

#### Resources and infrastructure

About half of the providers identified digital recordkeeping, limited availability of services/staff, overburdened specialists or funding issues as barriers to integrated care. A consumer participant noted that having no administrative support or a centralised case co-ordinator was a key barrier.

*The lack of action in addressing gaps in care [can be problematic]. Changes need to be made to the format to make it [care] accessible. They say sorry when things go wrong, but there is a lack of action* [Consumer 1 workshop 2].

Other consumers and providers noted that not having enough resources and staff in the emergency department to deal with issues in a MDT or enough specialists to cover all care would be problematic.

*The lack of resources for staff to provide care, to perform the checks and balances* [would be a barrier to care] [Consumer 2 workshop 2].

Several providers described barriers related to malfunctioning ICT and inadequate functioning EMRs, and not having contingency plans to enable access to patient notes. A provider noted that many of the ICT systems across the LHD are not fully integrated at present, so the transfer of information is limited.

#### Accessibility

Participants raised concerns about whether individual patient circumstances would be accommodated when accessing integrated care; for example, would the living situation of the patient be met? Many consumers concerned about systemic barriers to care, such as delays while waiting for the team and specialists to attend to them, poor consumer understanding of how to navigate care, patient mobility issues, poor consumer health or digital literacy, and lack of out of hours care. One consumer noted that culturally and linguistically diverse (CALD) patients with poor English proficiency may not receive the required assistance in navigating their care, language translation support, or guidance with treatment adherence.

Many consumers and providers noted that continuity of care issues could arise if there was insufficient integration among services and external providers. An example was accessing specialists for complex conditions if they were not physically co-located, especially if consumers need to move between locations to gain access to providers and there is a lack of transport and support services.

*Having an outsourced rather than inhouse team could present health risks to patients as they would not have timely access to care* [Consumer 1 interview 1].

A few consumers identified waiting to see the different members of the treatment team as an impediment to accessibility and expressed the feeling that that if not all the disciplines were available, they might need to go to another facility.

*The waiting is the hardest part. Everyone is busy. Waiting in the unknown (length of time) is hard* [Consumer 5 workshop 2].

Providers felt that a consumers’ lack of awareness and education about integrated care is limiting their utilisation.

*There are some integrated care models that already exist, but they are underutilised because [insert category] people don’t know about them* [Provider 4 workshop 2].

#### Patient factors and well-being

Consumers and providers described several patient factors that are important for integrated care to function well, such as the need for cultural safety, the need to be able to escalate care and ensuring that providers use a consistent approach to avoid gaps in patient care. Specifically, it was noted that some patients, especially those who are not confident with navigating escalation of care if needed, may not be suited to the model. Several providers felt that consumer choice was paramount and the model is not a fit for all.

*[The] social aspect of treating the elderly could be more challenging with this model* [Provider 2, workshop 2].

Some consumers were concerned about how integrated care would affect them if their health condition was unusual, if they live on their own, or they have a younger family. Risks were identified by providers for patients and staff if a co-morbid mental health condition is present. For example, if a patient’s social and psychological aspects of care are not attended to or the problem of provider workload and burnout are not addressed. One consumer expressed concerns about the focus of the model moving from the patient to profit.

*[I am concerned] if it [the model] becomes profit driven, rather than patient centred.* [Consumer 1 interview 1].

#### Safety and risks

Many consumers and providers expressed concerns about the potential for reduced quality of care if a poorly trained or impaired healthcare professional was part of the integrated care team. They also described potential risks if the information given to a patient was not consistent across providers, if delivery of treatment was not reliable, or if assessment was not adequate. A few consumers were concerned about documentation errors, posing safety risks.

*If records are not current and [the] system [is] not integrating* [Consumer 1 workshop 4].

Providers were concerned about patient privacy and potential litigation against providers. For example, many highlighted the risk associated with poor record keeping and information transfer, potential breaches of privacy, and the transmission of patient data via unencrypted emails, due to the sharing and transfer of patient information among multiple providers.

One consumer identified that integrated care may lead to a narrow focus for some providers; specialists may not look at the patient in a holistic manner, and only focus on the issue that concerns them. Another was concerned about inconsistent information given to the patients leading to poorer care.

*The lack of centralised support person, navigator or case worker [can create additional risk]. Without them, the patient gets lost in the system* [Consumer 3 workshop 2].

*People might get left in limbo [if care is inconsistent].* [Consumer 2 workshop 2].

## Discussion

**Fig. 3:**
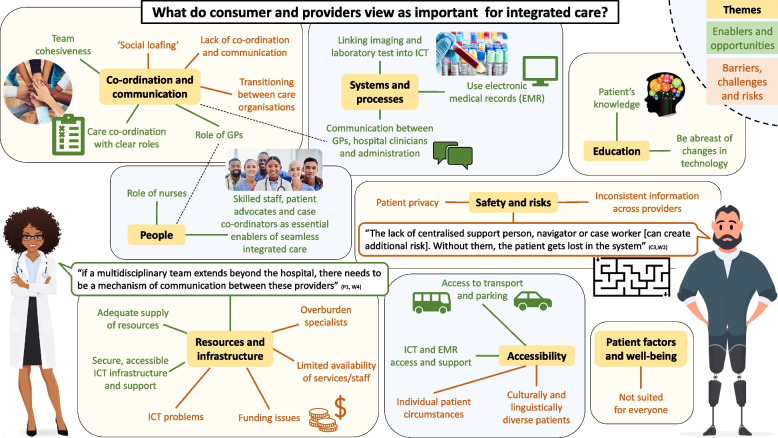
Rich graphic of integrated care

This study elicited consumers’ and providers’ needs and perspectives about integrated care from a diverse range of participants, to inform the design of a new metropolitan hospital. A rich picture, synthesising the main findings, are presented in Fig. 3.

Consumers and providers often talked about many of the same topics and themes but with different foci and emphasis. For example, similarities included concerns about a lack of after-hours support and the need for adequate resources and trained staff, communication pathways to be established, care to be patient-centred, the importance of patient and staff support in understanding the model, central care co-ordination, GP integration and involvement, and adequate infrastructure to support the model. Consumers felt that integrated care was a low risk, comprehensive and holistic model, that offers a level of reassurance for patients and their families. The providers described the model as able to cover a large group of specialities, reduce inpatient time, facilitate early and frequent team input, empower the patient and promote for clinicians the feeling of being a valued team member.

Consumers were concerned about not having an advocate or someone to help them navigate the integrated care system. They highlighted that clear communication, and the presence of a case co-ordinator would enable the model. These concerns are consistent with what has been reported in the literature about patient experience of integrated care: care co-ordination and integration between health and social care is central to ensure the model functions adequately [30]. Prior qualitative research confirms that patients highly value that all members of the care team are in harmony regarding their care [31]. Our study supports and expands upon these findings by providing the qualitative evidence that well co-ordinated care delivered by MDTs as part of an integrated and networked health service offers an optimal experience for consumers and providers. Providers were concerned about growing waitlists, poor continuity of care outside the hospital, lack of patient compliance, and poor record keeping if no one takes out of hours responsibility for the patient.

Integrated care is a well-accepted model. For example, the model has been associated with improvements in health-related quality of life, and improved patients’ confidence in the knowledge of their disease [21]. Positive effects have also been noted on physical and mental health outcomes [32]. Despite the evidence that integrated care has been implemented in a number of healthcare settings, there is a reported lack of clarity about the model’s concept and practice [33]. This is consistent with some of the themes that emerged in our study. Providers expressed concerns about a lack of staff accountability when there is no one with clear responsibility for the patient. Additionally, the providers noted the risks associated with staff not having a clear understanding of how the model works that is associated with poor communication and conflicting advice. Consumers felt that the model needed to undergo continual evaluation to ensure that it is valid.

The importance of communication among team members was a strong theme throughout. These findings demonstrate a level of awareness and investment in reducing the risks associated with communication gaps that have been associated with reduced interprofessional teamwork, compromised care, and distress [14–16]. The findings also support the notion that healthcare needs to be consumer focused and holistic [19]. Broadly speaking, these perceptions and concerns about integrated care are consistent with the strong theme of care-co-ordination described in a conceptual framework developed by Singer et al. [34].

Healthcare is shifting away from a standard hospital model to holistic, team-based approaches such at integrated care that also support wellness and keeping patients in their homes [1]. The insights reported in this study can be harnessed to improve integrated care in practice. For example, there was an emphasis on the central co-ordination of care, and safe networking of tertiary, primary and community services. Ideally, this could be complemented by other models such as virtual care and hospital in the home. Successful integration and interoperability of services and networks, requires an investment in resources and infrastructure to support systems that have the capability for providers to seamlessly access information along the pathway of integrated care.

## Study limitations

The following limitations should be considered when interpreting these findings: only one health condition (Type II diabetes) was presented in the workshop scenario. However, the model’s suitability for other conditions was discussed during the workshops and it was perceived to suit a diverse range of health conditions. Also, some participants may not have had any experience with integrated care, so their responses were hypothetical. The distribution of the participants was skewed toward specific characteristics. For example, most of the providers were nurses. However, this is reflective of roles within hospitals. Last, specific findings may not be generalisable to other health settings as consultations were conducted with participants of one LHD. In mitigation, the LHD in our study is a large one, servicing a demographically diverse population of approximately 300,000 residents [35] and comprises five large metropolitan public hospitals and associated care services.

### Study strengths and implications

This research is innovative and significant in the following ways: it is the first of its kind to utilise a unique design and methodology developed in collaboration with a hospital network. It used a data-driven approach to stratify the main diseases reported in the new hospital catchment as defined by the LHD. This research provides evidence, tailored to specific LHD demographics, that will help inform the development of a hospital of the future. Future research should focus on whether the enablers identified in the present study do lead to better care and model implementation, and if the perspective of patients receiving the model of care across a variety of health conditions would provide further insight into how the model works. Future studies should also focus on the implementation outcomes of integrated care that include extent of adoption and fidelity to intended practice using an established framework such as the Consolidated Framework for Implementation Research [36, 37].

## Conclusion

This study offers unique insights and evidence for the benefits, barriers, and enablers of the integrated model of healthcare delivery. Overall, the model was rated highly among participants, provided adequate resources, infrastructure, ICT systems, and processes are in place. Integrated care is a flexible and patient-focused model of healthcare, that offers significant benefits for a hospital of the future.

### Key learnings and relevance for integrated care

- The research is community focused and captured the views about integrated care from consumers and providers who currently reside in the catchment for a new hospital and will therefore be most likely to use the facility.
- Among the consumers and providers, common and divergent themes emerged. This is important as these relationships are central to the success of integrated care and addressing these could be paramount for improvements in model implementation.
- Fully integrating a primary care provider (e.g., GP) was valued by consumers and providers. This highlights one of the issues with Australian healthcare: GPs are funded on a national level, whereas hospitals are state funded. Addressing funding issues would improve the current fragmentation reported in healthcare [4].
- The findings could be harnessed to inform policies involving integrated care to establish feedback “checkpoints”. These would identify whether the strengths of the model are being realised and the barriers, such as patients falling through the cracks, avoided.

## Supplementary Information

**Additional file 1.**

**Additional file 2.**

**Additional file 3.**

## Data Availability

The datasets used and/or analysed during the current study are available from the corresponding author on reasonable request.
