# Supplementary material for: What do consumer and providers view as important for integrated care? A qualitative study
Source: BMC Health Serv Res. 2023 Jan 4;23:11. doi: 10.1186/s12913-022-08997-x (PMC9811795; doi:10.1186/s12913-022-08997-x)
Supplement: Supplementary file 2 — Additional file 2. [file 12913_2022_8997_MOESM2_ESM.docx]

**Provider: General Welcome**

“Thank you for coming along today and participating in our study. My name is XXX.

Acknowledgement of Country

The purpose of this workshop is to capture the needs of the community and their perspectives on new ways of delivering care. What we talk about today will inform the development of the new hospital. We really appreciate your time and thank you very much for being here today.

As providers of healthcare, your experiences as patients are very valuable. However, please note that there is no obligation to disclose details of personal healthcare issues.

Throughout the workshop a researcher/facilitator will be with each group to take notes, facilitate discussion and ask you questions. The workshop will go for 2 hours with a 10-minute break with refreshments after the first hour.

You will remain unidentifiable in the analysis and write-up of any findings relating to this research.

If you have any questions throughout, please ask one of the Macquarie University staff members (**introduce all staff members**).

Before we begin, we ask that you have read and signed the PICF.

**Individual Group Introduction**

“Good afternoon (morning/evening etc.) and welcome to the workshop. Thanks for taking the time to talk with us about what you would like to see in the new hospital. My name is (**insert name here**), and I am from the Australian Institute of Health Innovation at Macquarie University. My role as moderator will be to guide the discussion today.

Please note that there are no right or wrong answers but rather differing points of view. Please feel free to share your point of view even if it differs from what others have said. You don’t need to agree with others’ opinions, but we ask that you listen respectfully as others share their views.

We are taping the session because we don’t want to miss any of your comments. People often say very helpful things in these discussions, and we can’t write fast enough to get them all down. However, to make the recording as clear as possible, we ask that only one person speak at a time. And to remind you, no-one is identifiable on the recording.

1. **So that we can understand you a bit better, what is your discipline?**
2. **Are you mostly:**

Office based, facility based, a combination of both?

1. **What main problems do you currently encounter around delivering high quality care?**
2. **Can you suggest ways to overcome? (5 minutes)**
3. **What are you looking for in a new hospital?**

For example: IT/Scheduling/Accessibility

“Now that we have gotten to know each other a little better we would like you to reflect on a patient or patients who may or may not benefit from integrated care. We are interested in your thoughts about this model of care from your perspective and your patients’ perspective. The scenario describes a model you may have already encountered or engaged with. We would like you to think broadly.”

**Integrated Care**

Multidimensional needs of the patient are delivered in a coordinated manner by an interdisciplinary team or network of healthcare professionals.

Steve is a 70-year-old male with Type II diabetes who is obese and smokes a packet of cigarettes a day. He is having trouble walking so visits his local Emergency Department where he sees a co-located GP. The GP diagnoses a foot ulcer and identifies that Steve requires a full revision of his care. Steve will be looked after in hospital by a multidisciplinary team of healthcare professionals who share information and care (e.g., endocrinologist, ulcer team, nutritionist) using an electronic medical record system for communication.

Please answer the following questions:

1. **In an ideal world, how would his care be delivered?**

*Additional prompt*: how could you best model this?

**From your perspective:**

1. **How would this model help to solve the big problems for you?**

**(What are the pros/strengths for you?)**

1. **What barriers limit this model for you?**
2. **What enablers would need to be in place for this to work?**

**From your patients’ perspective:**

1. **How would this model help to solve the big problems for your patients?**
2. **What might be the pros/strengths?**
3. **What barriers might limit this model for your patients?**
4. **What enablers would need to be in place for this to work?**

**General questions:**

1. **What proportion of your patients would this model work for?**

Low – Mid – High

1. **Can you think of anything about it that might be impractical?**
2. **Can you think of anything about it that might be unachievable?**
3. **To what extent could this model be applicable to other health conditions? What conditions?**
4. **Can you think of any clinicians or patients who might find this model of care difficult to access?**
5. **Is there anything about the model that concerns you?**
6. **What might be the safety issues for your patients?**
7. **Do you see any risks to you as the healthcare provider?**

*(Additional prompts)*

*Are there any potential risks that you can identify?*

- - *“Why is that?”*
  - *Can you suggest a better way?*

**Concluding remarks:**

We will be looking at your data to find commonalties between providers.

We are conducting exploratory research to gather information only. Thank you for your time.
