# Supplementary material for: What do consumer and providers view as important for integrated care? A qualitative study
Source: BMC Health Serv Res. 2023 Jan 4;23:11. doi: 10.1186/s12913-022-08997-x (PMC9811795; doi:10.1186/s12913-022-08997-x)
Supplement: Supplementary file 3 — Additional file 3. [file 12913_2022_8997_MOESM3_ESM.doc]

Thank you for agreeing to allow us to contact you for a follow up call to talk about the findings of the discussions. The purpose of this phone call is to expand on areas of interest and verify the findings from the workshop data. What we talk about today will continue to inform the development of the new hospital in Rouse Hill. The discussion will be recorded so we can capture everything that is said.

You are free to withdraw at any time without consequence.

Do you have any questions before we start? (pause for questions).

For integrated care, can you please elaborate on what you think is meant by….
